# A Comprehensive Review of Pathological Examination in Forensic Medicine: Past, Present, and Future

**DOI:** 10.7759/cureus.22740

**Published:** 2022-03-01

**Authors:** Dezy Singh, Ramesh Chand Tiwari, Arvind Kumar, Ashish R Bhute, Ravi P Meshram, Manisha Dikshit, Ved Bhushan Sharma, Bhawana Mittal

**Affiliations:** 1 Department of Agad Tantra Evum Vidhi Vaidyaka, Uttarakhand Ayurved University (UAU) Rishikul Campus, Haridwar, IND; 2 Department of Pathology and Laboratory Medicine, All India Institute of Medical Sciences, Rishikesh, Rishikesh, IND; 3 Department of Forensic Medicine and Toxicology, All India Institute of Medical Sciences, Rishikesh, Rishikesh, IND

**Keywords:** progress notes, ancient, forensic expert, from india, resource limited settings, pros, forensic pathologist, histopathology examination, forensic pathology, autopsy

## Abstract

Pathological examination (PE) encompasses a gross or macroscopy and histopathological or microscopic examination. It is prudent in finding the cause of death (COD) in clinical and medicolegal autopsies. There are various auxiliary techniques in the form of clinical history, communication, specialized training, and protocols for consolidation of the PE results. After a thorough search of the literature in PubMed with relevant keywords along with further analysis of the results, it emerged that even with the modernization of forensic medicine, a PE is unbeatable in detecting the COD. It has various useful aspects, apart from regular finding the COD, such as in student teaching, epidemiology of disease, audit tool, and quality assurance. There are also limitations of PE, which should be dealt with great caution. Hence, limitations must be understood by a forensic expert as well as a pathologist. In this review, all factors that are related to PE in any manner are discussed in detail, and the scope for improving the quality of PE to be relevant in the present scenario is reviewed. It is a comprehensive reassessment of the literature review that also casts light on the future along with a critical analysis of the facts that deal with PE.

## Introduction and background

An autopsy has been practiced since ancient times. With the evaluation of time, it also got shaped up by giants of this field from different parts of the world, and it emerged with multifaces as a part of medical sciences, which not only tells about the cause of death (COD) but also about the pathogenesis, demography of disease, as a part of medical teaching, helping in acquiring new preventive measures of disease, and retrospective quality assessment of clinical diagnosis [1-5]. Many unanticipated pathological findings challenge clinicians to explore the horizon of medical sciences. Pathological examination (PE) is an integral part of clinical and medicolegal autopsies. Microscopic and gross findings along with relevant clinical history are the key to ascertaining post-mortem COD.

Communication between a forensic expert and a pathologist is an important step in making the post-mortem diagnosis without misunderstanding [6-8]. A meticulous training is a must for all those who perform the clinical or medicolegal autopsy (MLA), and it is also necessary for a pathologist to get the verse in PE in the autopsy that includes grossing, microscopy, and interpretation since this is quite different from regular surgical pathology [9]. There are guidelines for the uniformity of standard operating procedure (SOP) to conclude the COD. Adherence to laid down protocols and guidelines of autopsies is mandatory for all autopsy practitioners. Forensic experts and pathologists must know the limitations of PE, which hinder the post-mortem COD.

Clinical autopsy in India is not frequently done except at a few large tertiary centers due to the social stigma as relatives are not ready to give consent. Not all medicolegal autopsies are routinely subjected to histopathologic analysis. Only those cases where the COD is either not readily apparent or obvious on gross autopsy are sent for histopathologic examination. Many authors are in favor of gross examination when microscopy is inconclusive [10,11]. In an MLA, only representative tissue of certain organs is taken. Whole single organs are seldom sent for PE. A systematic and thorough tissue sampling is necessary for MLA, and this practice is usually not followed even in developed countries [12]. This study is directed to find all the possible pros and cons of PE in the autopsy as well as to search the limitations and correcting measures. This study also points out the progress of medical autopsy within a span of time.

## Review

Materials and methods

A literature search was performed only in PubMed using the following terms: pathological examination, autopsy, forensic medicine, histopathology, gross examination, limitations, improvement, clinical and medicolegal autopsy, history, and India. Articles were reviewed for relevance and included if they contained information about the PE with pros and cons and the scope of improvement in clinical and medicolegal autopsy cases with no time bars. A total of 3,875 source results were obtained after searching for the keywords either alone or in combination, with no time bar. After refining the search criteria, 927 sources showed up as results. Out of 927 sources, only 62 were integrated into this review article as references due to the maximum suitability of the article. The reference lists from each article were reviewed for other relevant material. This information was synthesized and included in this review.

Historical aspect

The history of the autopsy covers a very long period as it starts from before Christ (BC) to date. The word “autopsy” was derived from the Greek words “autos” (self) and “opsis” (eye), loosely translated as “seeing for oneself.” The Alexandrian scientists, Herophilos (335-280 BC) and Erasistratus (304-250), were the first to dissect the human body [13].

In ancient India, Sushruta (600 BCE) and Charaka (400 BCE) were some of the renowned medical practitioners of ancient India that started the surgery for the cure and as a learning tool of anatomy and function of organs [14]. They mainly sacrificed the animals and, to a lesser extent, improperly buried bodies for this purpose, and examinations of patients by physicians [15].

The book “Kautilya’s Arthashastra” describes the necessity of autopsy in establishing the COD. It states that the magistrate shall conduct a post-mortem on any case of sudden (unnatural) death after smearing the body with oil to bring out bruises, swellings, and other injuries. Kautilya’s Arthashastra is a monumental work written by Kautilya in 400 BC [16]. The first MLA in India was performed on August 28, 1693, when James Wheeler (Member of Council, Sea Customer and Chief Justice of Choultry) died in Chennai (formerly Madras) [17]. The three studies with the largest number of autopsies (one with 40,130, a second with 25,066, and a third with 14,074 patients) are from the United States [18-20]. The progress of pathology in India during the British Raj began with the establishment of the first Bengal Medical School in 1822 in Calcutta (now Kolkata), which was converted into a Bengal Medical College (now Kolkata Medical College) in 1835 [21]. Another report from the Post Graduate Institute of Medical Education and Research (PGIMER), Chandigarh, describes a comparative analysis of 1000 autopsies from 1982 to 1987 [22]. A study by Lanjewar et al. is the fourth largest study in the world and first in India, which unfolds the various aspects of autopsy from specimen preservation to updating of autopsy technique [2].

Significance of PE

PE (gross and histopathological) has many implications beyond detecting the COD. It is used to confirm the gross/macroscopic changes that may lead to death by microscopic examination [5,23] as an alternative to gross examination when gross morphological findings are not yielding results [9] to confirm the antemortem clinical diagnosis and ascertain the demography of a certain disease [24-26]. Unusual findings can only be noted after the PE in a form of permanent documentation of pathologies identified at autopsy [2]. An autopsy is mandatory for retrospective quality assessment of clinical diagnosis [27] as an essential source of material for medical undergraduates and postgraduate teaching [28].

Elements of PE

Role of Gross (Macroscopic) Examination in Autopsy

The term "grossing" means inspecting the specimens, describing and measuring the tissue, inking if needed, and sectioning the tissue to be processed for diagnosis. The examination of organs and tissues macroscopically to establish a diagnosis and select relevant portions for subsequent microscopic examination and special studies is fundamental to the practice of pathology [9]. The macroscopic examination is being practiced in forensic autopsy far earlier when microscopy was in the early phase as called by some authors in the pre-histology era (1950).

Many studies advocate the superiority of macroscopic examination over microscopic examination on arriving post-mortem diagnosis. According to Geller et al., 90% of diagnoses can be made by an experienced pathologist by only seeing the gross pathology of the organ [9]. Chatelain et al. stated that microscopic examination must be performed in certain organs only [10]. Molina et al. and Takatsu suggested that macroscopic examination can make microscopic finding interpretation easy, but histology is reserved for certain organs only [11,12]. Man et al. did not find any histopathological abnormality in grossly normal-looking internal organs in case of stillbirth and intrauterine death [29]. de la Grandmaison et al. noted that histopathology is not helping to ascertain the COD; only in 8.4% of cases, the COD was established by histopathology [30].

Contrary to that, many studies are in strong favor of M/E rather than totally relying on gross findings [31-34]. Thomas et al. concluded that a thorough histological examination (≥20 histological sections) is a strong predictor of incidental in-situ cancer and atypical hyperplasia in the breast [34]. Rohan et al. declared that microscopy is the definitive diagnosis in neurodegenerative disorder that imparts invaluable feedback to clinicians as well as biochemical and radiological diagnostics [35]. de Noronha et al. also disregarded the whole importance of gross examination of heart findings as most of the young sudden cardiac death patients showed grossly normal heart (45%), and only 10% exhibited coronary artery disease [36]. Mohorea et al. were able to put their findings only based on microscopy as grossly, the thyroid glands were normal [37,38].

Role of Histopathology in Autopsy

Histology is used in forensic practice as an ancillary investigation to investigate cases in which the macroscopic examination has failed to identify the COD [39]. Many studies in clinical and MLA autopsy cases declared the unparalleled role of PE including gross examination and microscopic examination to find the COD or pathogenesis or to confirm the clinical diagnosis. Roulson et al. concluded that histopathology is still the most accurate method for determining the COD and auditing the accuracy of clinical diagnosis, diagnostic tests, and death certification [23]. Lanjewar et al. pointed out that PE in autopsy plays an important role in providing information about conditions that are no longer prevalent and also advocate the role in ascertaining the COD [2]. Schäfer et al. and Höflmayer et al. pointed out that sets of pathological findings in autopsy in different eras may provide clues to the changing spectrum of disease [24,25]. Zaitoun et al. concluded in their study that autopsy histology is the most accurate method for determining the COD and auditing the accuracy of clinical diagnoses [5].

Start et al. are in strong disagreement with the suggestions that advances in diagnostic techniques have diminished the role of autopsies [28]. PE in autopsy is essential for retrospective quality assessment of clinical diagnosis by Kurz et al. [27]. Histopathological diagnosis on autopsy correlated well with the clinical COD by Khare et al. [1]. The autopsy, if combined with relevant details and histopathological examination, is of great value in establishing reasons that led to death. Todorović et al. stated in their study of lung autopsies in illicit drug users that histopathological study is necessary to determine a COD when a deceased person has a history of dependence or abuse of psychoactive drugs with negative toxicological results [40].

In a study by Delteil et al., histopathological examination in 70% of cases highlighted that forensic histopathology is an indispensable tool in pediatric medicolegal autopsies [3]. Zhou et al. believed that PE is essential for an autopsy to understand the pathogenesis and ascertain etiology [4]. According to Scheimberg et al., fetal PE is still the gold standard in the diagnosis of fetal abnormalities [41]. According to Raghuram et al. and Shanmugasundaram et al., autopsies with histopathological diagnosis continue to be important for improving the diagnostic insight and may improve future clinical care and is the gold standard even in the era of modern imaging technology [42,43].

Recently, a large study conducted by Becas et al. in France concluded that pathological expertise was required in 19.2% of 630 MLA cases [44]. The pathologist's expertise enabled to change the COD in 22% of cases and the manner of death in 19%, whereas the expertise did not help in cases of homicides, suicides, and accidents. There is a critical role of histopathology in narrowing down the discrepancy between antemortem and post-mortem diagnoses (Table 1).

**Table 1:** Discrepancy between antemortem and post-mortem diagnoses after histopathological finding

S. No.	Authors	Year	Study group	Discrepancy rate	Cause
1	Roulson et al. [23]	2005	Meta-analysis, 1960-2005	20%	Pulmonary embolism and cardiac cause
2	Lanjewar et al. [2]	2018	Retrospective study 2003-2012	31%	Infection and cardiac cause
3	Raghuram et al. [42]	2021	Pediatric cancer patient	10%	Infection and missed cancer diagnosis
4	Pastores et al. [45]	2007	Critically ill patient	26%	Opportunistic infection and cardiac cause
5	Zaitoun et al. [5]	1998	Hospital admits, cases died	23%	Pulmonary embolism, bronchopneumonia, and ischemic heart disease
6	Costache et al. [46]	2014	Retrospective study	24%	Pulmonary embolism
7	Delteil et al. [3]	2018	Infant autopsy	30%	Infection

In Table 1, the discrepancy rate varies in a range from 10% to 30% depending on autopsy number and organ of interest. The majority of authors are in favor of infection followed by pulmonary embolism and cardiac causes. These causes are the principal etiological factors in decreasing order that led to the discrepancy [2,3,5,23,26,45,46].

Unusual histopathological findings

One of the important aspects of PE is the detection of incidental and unusual pathological lesions that contribute to an enhanced understanding of the pathogenesis and epidemiology of the disease. It also helps in directing the formulation of the screening program. There are several studies by Nakajima et al., Becker et al., Amarapurkar et al., Berry, and Nayak et al., which proved the practical execution of PE in this field that contributed to the medical sciences [47-51] (Table 2).

Significance of clinical history in pathological examination

**Table 2:** Unusual findings revealed on histopathology

S. No.	Authors	Year	Study group	Most common histological findings	
1.	Nakajima et al. [47]	2011	Sudden cardiac death cases without coronary atherosclerosis	Concluded about Pokkuri death syndrome.	
2.	Becker et al. [48]	1976	Intrauterine death, hospital stay	Increased fat deposition within fetal adrenal glands leads to fetal physiological stress.	
3.	Man et al. [29]	2016	Stillbirth and intrauterine fetal death	A majority of internal organs are normal on both macroscopic and microscopic examination.	
4.	de Noronha et al. [36]	2014	Young sudden cardiac deaths	Mainly morphologically normal heart followed by cardiomyopathy.	
5.	Amarapurkar et al. [49]	2005	Liver in HIV patients	Non-specific tuberculosis is the commonest infection.	
6.	Patel et al. [26]	2016	Retrospective study of 269 cases	Atherosclerosis was followed by fatty liver.	
7.	Berry [50]	1992	Pathological findings in SIDS	Subserosal petechial hemorrhages and mild fatty change in the liver.	
8.	Mohorea et al. [37]	2021	Retrospective autopsies of 526 cases	Papillary microcarcinoma.	
9.	Shanmugasundaram et al. [43]	2020	Fetal autopsy of 177 cases	Septal defects (45%) were the most frequent cardiac defect followed by left ventricular outflow tract obstruction.	
10.	Nayak et al. [51]	2016	Fetal autopsy of 255 cases	Renal anomalies were noted in 40% of cases.	

Many studies are in favor of knowing clinical history before doing the PE that paves the path to detect the COD. There is concordance among many researchers that if clinical history is supplemented with a histopathological examination, then it can facilitate the post-mortem diagnosis to identify the cause that led to death [1,6,31,52,53].

Significance of communication between forensic experts and pathologists in PE

The communication gap between pathologists and forensic experts may be one of the causes that can easily hinder the exact COD in autopsy. Hence, forensic experts and clinical pathologists must establish robust working relationships in a cooperative environment. And this fact is supported by a majority of researchers by their studies [6-8,34].

Need for specialized training

Lack of appropriate training of pathologists and forensic experts always widens the range of discrepancy of diagnosis. Geller et al. along with many authors strongly recommended pathologists to improve the quality of PE [7-9,54,55]. Royal College Pathologist, European Society for Forensic Science, and American Academy of Forensic Sciences laid down various guidelines/protocols for death in different death scenarios with continuous training programs in the wake of updating and uniformity. A study by de Noronha et al. is in favor of expert cardiac pathologists that certainly improve the accuracy [36]. Hansen stated that regular autopsy does not reveal the desired findings; hence, it needs to adjunct with clinical history and protocol-based grossing for PE of heart in autopsy cases [7]. Hansen, Kurz et al., and Basso et al. supported the protocol-based forensic pathologist training [7,27,56].

Role of MLA

A systematic and thorough visceral organ examination macroscopically and microscopically with precise autopsy protocol is essential in MLA, while in clinical autopsy, representative tissue samples can be taken [3,12,33]. In MLA, gross examination with description and representative section of tissue from an organ needs meticulous training and expertise [9].

Protocols/guidelines of autopsy

Protocols/guidelines are the guiding paths that are specified for certain death scenarios irrespective of the nature of the autopsy, whether medicolegal or clinical. Various protocols in MLA are laid down by the Royal College of Pathologists with different PE approaches. Association for European Cardiovascular Pathology (AECVP) produced a guideline for the post-mortem investigation of patients who die suddenly from cardiac diseases [56]. Apart from this, there are protocols for clinical autopsy, which direct the PE [57]. Failure to adhere to the guidelines will create a high chance of missing the COD. Hence, Kotabagi et al. further increased the hurdle in the post-mortem diagnosis, and more sophisticated training is required for MLA [32]. So, autopsy in the absence of specified protocol led to a great possibility of missing the COD.

Hindering factors and correcting measures

Several factors have been mentioned by many authors regarding PE that poses the difficulty of arriving at the post-mortem COD [7,10,31,53]. Better communication between forensic clinicians and pathologists is vital for hustle-free work. Clinical history is invaluable if combined with histopathological findings to rule out the misdiagnosis. Banner et al. admitted that only 50% of forensic experts follow the standard protocol due to the lack of training [55]. Madadin et al. are in favor of regular training of staff for updating SOPs to execute the best [53]. de Noronha et al. mentioned organ-specific training to find any hidden pathology [36]. Thomas et al. concluded in their study that the number of biopsy samples is crucial to pick up the precursor lesion in the breast. Hence, they have given a problem statement and solution at the same time [34]. There are issues with the funding, infrastructures, and disparity between medical authorities and administration in many countries, especially in developing countries, which grossly affect the quality and outcomes of autopsy procedures [1,55,58]. The lack of a uniform system in forensic medicine creates difficulties in assessing the development and performance of forensic medicine as a distinct discipline [59]. The creation of autopsy pathology as a subspecialty might also solve the issue of late and insufficient reporting [13].

Current status in India

According to Das Gupta et al., the time lag interval between the discovery of the body and the autopsy is often very long in India [60]. Facilities of histopathological examinations of the autopsied tissues are available only in a small number of institutions in India. In addition to that, Khare et al. discussed that inadequate sampling, improper fixation, and failure to send representative sections led to misdiagnosis [1]. The National Medical Commission (NMC) does not have any provisions for inter-departmental training or postings in the pathology department of forensic and toxicology (FMT) for postgraduate residents. A study by Yadav J et al. described the prominent limitations in the field of autopsy in India [58]. According to their study, obtaining consent is the biggest obstacle, and the consent could be obtained only in 15% of COVID-19-related death cases. In India, the most underrated area of hospital/institute is mortuaries. In many places, they even lack the basic infrastructure to carry out routine non-infectious autopsies. Hence, there is a need for the development of customized protocols in each setup, considering the high risk of infection in conducting autopsies. Another limitation is in the form of a non-performing autopsy by a pathologist like in western countries. Here in India, only forensic experts do the autopsy procedure [58].

There are great prospects of PE even with a few limitations besides regular clinical and medicolegal autopsies such as finding out the COD in clinically unsuspected pathology even in the current era of “high-tech medicine” [13,61]. It has also got value in clinical, educational, and epidemiological fields [62]. In this review, we have attempted to touch every aspect of PE in an autopsy that directly or indirectly affects the COD and broadens the insight of forensic experts and pathologists.

## Conclusions

PE is the gold standard in ascertaining the COD despite the modernization of techniques in autopsy. It has an indispensable role in ascertaining the epidemiology of disease with clinical and educational values for medical students, making it irreplaceable in the future. There is a great scope of improvement in the PE as regularly specialized training is a must for both the forensic experts and pathologists for clinical and medicolegal autopsies. A protocol-based PE is indispensable and always leads to the right direction of the autopsy examination. Communication between forensic experts and pathologists is an essential tool for arriving at the post-mortem diagnosis. From ancient to modern time, pathological examination has remained one of the most important branch of medicine.
